# Knowledge, Readiness, Willingness-to-Use, and Willingness-to-Pay for Telehealth in Nonlife-Threatening Emergency Department Visits

**DOI:** 10.1089/tmr.2024.0085

**Published:** 2025-01-27

**Authors:** Vahé Heboyan, Phillip Coule, Davide Mariotti, Gianluca De Leo

**Affiliations:** ^1^ Augusta University, Augusta, GA, USA.; ^2^ Walden University, Minneapolis, MN, USA.

**Keywords:** emergency medicine, telehealth, willingness-to-pay, patient perception

## Abstract

**Background:**

The emergency department (ED) provides a significant portion of health care services in the United States, and its utilization has increased over the past decade. ED overcrowding remains a considerable challenge to many EDs. The objectives of this study were (1) to evaluate the knowledge of telehealth and readiness to use it among patients who visit EDs in a nonurgent triage category and (2) to estimate their willingness-to-use and willingness-to-pay for telehealth consultations.

**Methods:**

A structured questionnaire was administered using a tablet to adult patients who visited the ED of a large medical center and who were triaged into a nonurgent category. Respondents were asked about their sociodemographic and ED visit characteristics and health and telehealth utilization history. Then, we presented them with a hypothetical scenario for visiting a board-certified ED doctor through telehealth instead of in-person visits, and, using a double-bound dichotomous choice iterative bidding algorithm, we solicited their willingness-to-pay for such a telehealth visit.

**Results:**

A total of 171 patients agreed to participate in the study. More than half of the respondents (*n* = 107; 62.6%) said they have health insurance. Almost half of the respondents (*n* = 71; 41.5%) reported the main reason for going to the ED was an ongoing condition or concern. More than two-thirds of the respondents identified themselves as being very proficient with using a smartphone or tablet (*n* = 116; 67.8%), and only a few (*n* = 21; 12.3%) reported not having any internet-capable device. Most respondents (*n* = 148; 86.5%) had never heard about telehealth. However, after a brief description of telehealth, we found that approximately two-thirds of the patients would be willing to use or consider using telehealth (*n* = 107; 62.6%), and one-third (*n* = 64; 37.4%) would not be interested. We did not observe any statistically significant differences in willingness-to-use. However, we observed statistically significant differences in the willingness-to-pay $50 by gender (*p* < 0.01), by currently having a regular doctor/clinic (*p* < 0.05), and by health insurance status.

**Conclusions:**

Hospitals should consider investigating telehealth services that can be provided to their communities as an option instead of visiting their EDs. While technology does not seem to be a barrier to telehealth, more educational initiatives to inform the public about telehealth are desirable. A targeted advertisement campaign to recommend telehealth for nonlife-threatening ED visits could be developed once more user characteristics are collected.

## Introduction

In the United States, emergency departments (EDs) provide significant medical care, with an estimated 151 million total ED visits occurring in 2019.^1^ About 22% of adults aged 18 years and over had visited the ED at least once in the past 12 months.^1^ The number of patients using the ED is increasing, and the majority of these patients constitute nonemergency patients.^2^ Patients decide to use EDs because alternatives are complex due to a general lack of outpatient services, inadequate primary care capacity, unavailability of after-hours care, and lengthy waits for primary and specialty care appointments.^3^ The impact of ED crowding on mortality, morbidity, medical error, staff burnout, and excessive costs is well documented.^3,4^ It has also been reported that the number of patients with nonurgent complaints makes emergency care services and procedures complex.^5^

The Health Resources and Services Administration defines telehealth as using electronic information and telecommunications technologies to support long-distance clinical health care, patient and professional health-related education, public health, and health administration.^6^ A recent research study found a negative correlation between the amount of care family physicians provided virtually and their patients’ ED visits.^7^ Several articles have highlighted the benefits of using telehealth to support EDs: telehealth implemented in skilled nursing facilities allows residents to remain in place for treatment rather than visiting the ED;^8^ a telehealth emergency service for high-risk heart failure patients is safe and reduces unplanned hospitalizations;^9^ professional sexual assault nurse examiners remotely assist nurses from smaller hospitals or rural communities with sexual assault response examinations to provide a thorough, comprehensive examination of patients;^10^ and diagnostic telehealth consultations of low-risk patients with acute respiratory symptoms are not inferior to face-to-face evaluation at ED.^11^ A study examining ED nurses’ perceptions and experience with telehealth reported that nurses felt optimistic regarding telemedicine, believing it would significantly improve patient outcomes.^10^ During the COVID-19 pandemic, a telehealth system managed >800 patients daily and prevented about half of the patients from visiting an ED or urgent care in person.^12^

While there is considerable literature on the efficacy and the adoption of telehealth, there has been little work on patients’ knowledge of telehealth and on their willingness-to-use and willingness-to-pay for telehealth services that target EDs. The objectives of this study were (1) to evaluate the knowledge of telehealth and readiness to use it among patients who visit EDs in a nonurgent triage category and (2) to estimate their willingness-to-use and willingness-to-pay for telehealth consultations.

## Methods

### Participants

Data were collected at the Emergency Department of the Augusta University Medical Center (AUMC), located in Augusta, GA, USA. AUMC is an urban 500-bed tertiary hospital that provides comprehensive primary, specialty, and subspecialty care in the region. Specialized services include the region’s best-equipped and busiest Level I Trauma Center, serving patients from over 13 counties, and specialized transplantation for pancreas and kidney. AUMC is the primary teaching and patient care site for the Medical College of Georgia.

The ED is a 40-bed facility that provides emergency medical services to more than 76,000 patients annually. The emergency severity index (ESI) is a five-level ED triage algorithm that provides clinically relevant stratification of patients into five groups from 1 (most urgent) to 5 (least urgent) based on acuity and resource needs (Agency for Healthcare Research and Quality). To be included in the study, subjects had to be adult patients (>18 years) who were waiting at the AUMC ED and who were triaged into levels 3, 4, and 5 of the ESI scale,^13^ spoke English, consented to participate in the study, and were able to use a tablet without assistance. This study was approved by the Institutional Review Board of Augusta University (ID# 1082478).

### Data collection process

During the Fall 2017, two graduate students volunteered to collect data at the AUMC ED on different weekdays between 11:00 a.m. and 8:00 p.m. The ED staff identified patients in triage levels 3, 4, or 5. Students located the patients in the ED waiting area, introduced the study, and asked them to participate voluntarily. Once the participants consented to the study, they were asked to complete the survey questions participants using a tablet. The survey process was terminated if patients were called to the ED examination rooms or did not feel well enough to continue answering questions.

### Survey design

**FIG. 1.**
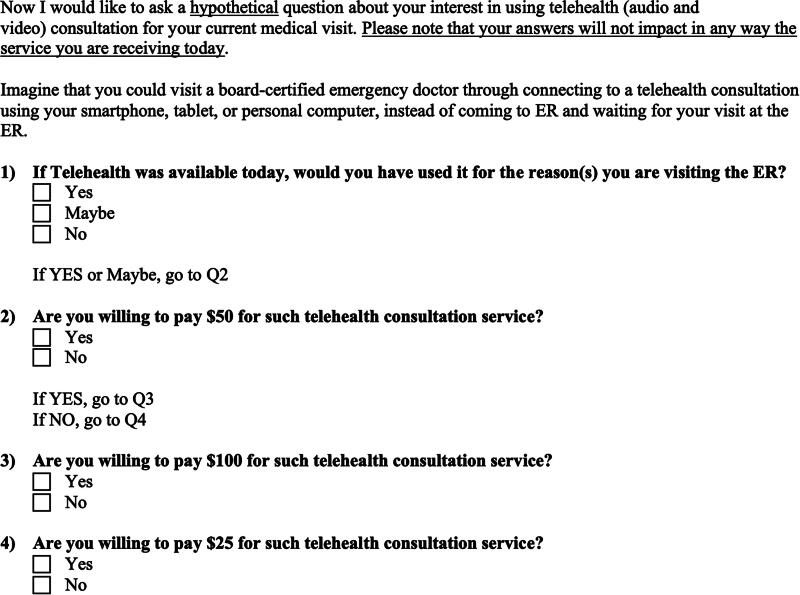
Double-bound dichotomous choice survey questions.

Based on feedback from subject matter experts (ED physicians and telehealth scholars), we developed a survey using Qualtrics^XM^ that consisted of six domains. The demographic domain collected information about age, gender, race, level of education, employment, married status, and the number of children under 18 living in the same household. The second domain focused on the visit to the ED and collected the main reasons for such visit, the distance in miles the subjects had to travel to reach the ED, the time spent waiting to be processed, and the reasons why the ED was chosen among other EDs in the area. The third domain focused on the subjects’ health and insurance status. It asked questions about the number of times the subjects saw a general practitioner during the previous 30 days and the number of times they visited an ED in the last 30 days. The third domain also asked about the type of health insurance the subjects had and who would be responsible for paying for the ED visit. Participants were also asked to rate their overall health, not counting the current main health problem that brought them to the ED. The fourth domain collected information about smartphone/tablet ownership and technical literacy. The fifth domain focused on gathering information on knowledge and past use of telehealth and questions related to the subject’s interest in using telehealth for future nonemergent medical needs. To understand the patient’s approval and perceived value of the telehealth consultation for emergency medical needs, the last domain on the survey first offered an explanation of an hypothetical audio and video telehealth visit and then asked subjects a set of questions to determine their willingness-to-use and to estimate their willingness-to-pay for telehealth consultation using the double-bounded dichotomous choice (DBDC) contingent valuation method (CVM). In the DBDC CVM, respondents are first asked if they are willing to pay a specific amount for a good or service. Based on their response, a follow-up question is asked with a higher or lower amount. This helps in narrowing down the range of the respondent’s willingness-to-pay. This method provides more information per respondent, improving the efficiency of statistical estimates.^14,15^ We first described a hypothetical scenario where a patient could access a board-certified emergency doctor through telehealth consultation instead of coming to the ED and waiting for the service. Then, subjects were given the initial willingness-to-pay question, which asked if they would be willing to pay $50 for the described service. If subjects responded “yes” to this initial question, they were asked a follow-up question where the monetary amount was raised to $100. If subjects responded “no” to the initial question, they were asked the follow-up question with a lower monetary amount of $25. At the time of this project, we determined that several telehealth services that allowed people to connect with a physician were available on the market as an out-of-pocket expense, ranging from $50 to $100 for each encounter. Figure 1 shows our DBDC survey questions. The survey took <15 min to complete. Subjects were allowed to skip questions.

### Statistical analyses

We start the analyses by presenting patient sociodemographic and ED visit characteristics, healthcare and telehealth utilization history, and willingness-to-use telehealth. Data are given for the whole sample and by gender and race. Statistical tests (e.g., Chi-squared or Fisher’s Exact) are used to test if statistical differences exist in data by gender and race. We describe the double-bound dichotomous choice survey questions and their results. We followed the methodology and iterative bidding algorithm in previous research studies^16,17^, which presents a hypothetical scenario and solicits respondents’ willingness-to-pay for visiting a board-certified ED doctor through telehealth. All analyses are done in Stata 16.^17^

## Results

A total of 171 subjects agreed to participate in the study. More than half of the respondents (*n* = 103; 61.3%) belonged to category 3 of the ESI scale. There were 60 (35.7%) respondents in category four and five (3.0%) in category 5. Incomplete questionnaires were not considered in this study. In some cases, participants were not able to complete the questionnaire because they were called for their visit. We did not include these partial responses in our analysis. We considered all the participants who completed the questionnaire. Two participants skipped few of the demographic questions.

### Respondents’ characteristics

Two-thirds of the respondents were female (*n* = 107; 62.6%). The two most common races were black (*n* = 106, 62.0%) and white (*n* = 51, 29.8%). Respondents were distributed among the following age categories: 18–25 years old (*n* = 30; 17.7%), 26–35 years old (*n* = 48; 28.2%), 36–45 years old (*n* = 37; 21.8%), and over 46 years old (*n* = 55; 32.4%). The education level reported was less than high school (*n* = 25; 14.6%), high school or GED (*n* = 75; 43.9%), and more than high school (*n* = 71; 41.5%). Respondents were single (*n* = 91; 53.2%) and married or with a partner (*n* = 76; 44.4%). Approximately half of the respondents were employed (*n* = 75; 43.9%). Less than half (*n* = 75; 43.9%) of the respondents reported having no children under 18 living in the same household. More than half of the respondents (*n* = 107; 62.6%) said they have health insurance. The top two types of insurance were Medicaid, Medicare, or other government insurance (*n* = 62; 36.3%) and health insurance at work provided by employers (*n* = 32; 18.7%). Slightly more than one-third of the respondents (*n* = 64; 37.4%) reported being uninsured. Respondents rated their overall health, not counting the health problem that brought them to the ED, as poor (*n* = 14; 8.2%), fair (*n* = 49; 28.7%), good (*n* = 44; 25.7%), very good (*n* = 39; 22.8%), and excellent (*n* = 25; 14.6%). We observed statistically significant differences for race by education (*p* < 0.05), marital status (*p* < 0.05), employment status (*p* = 0.06), triage level (*p* < 0.05), and gender by number of children (*p* = 0.09). Table 1 provides detailed information on the characteristics of the respondents.

**Table 1.** Respondents’ Characteristics

Variable	*N*	%	Female %	Male %	White %	Black %	Other %
Gender							
Female	107	62.6			60.8	65.1	50.0
Male	64	37.4			39.2	34.9	50.0
Race							
White	51	29.8	29.0	31.3			
Black	106	62.0	64.5	57.8			
Other	14	8.2	6.5	10.9			
Age							
18–25	30	17.7	20.8	12.5	15.7	17.1	28.6
26–35	48	28.2	26.4	31.3	21.6	31.4	28.6
36–45	37	21.8	20.8	23.4	21.6	22.9	14.3
over 46	55	32.4	32.1	32.8	41.2	28.6	28.6
Education (*p* < 0.05)							
Less than High School	25	14.6	14.0	15.6	**21.6**	**8.5**	**35.7**
HS or GED	75	43.9	40.2	50.0	**39.2**	**50.0**	**14.3**
More than HS	71	41.5	45.8	34.4	**39.2**	**41.5**	**50.0**
Marital Status (*p* < 0.05)							
Single	91	53.2	50.5	57.8	**33.3**	**65.1**	**35.7**
Married/Partnered	76	44.4	47.7	39.1	**62.8**	**33.0**	**64.3**
Unknown	4	2.3	1.9	3.1	**3.9**	**1.9**	**0.0**
Employment (*p* = 0.06)							
Employed	75	43.9	39.3	51.6	**31.4**	**50.9**	**35.7**
Unemployed	40	23.4	26.2	18.8	**29.4**	**17.9**	**42.9**
Other	56	32.8	34.6	29.7	**39.2**	**31.1**	**21.4**
Number of children (*p* = 0.09)							
0	75	43.9	**40.2**	**50.0**	54.9	37.7	50.0
1	35	20.5	**19.6**	**21.9**	13.7	23.6	21.4
2	29	17.0	**22.4**	**7.8**	15.7	16.0	28.6
3+	32	18.7	**17.8**	**20.3**	15.7	22.6	0.0
Insured							
No	64	37.4	28.0	53.1	45.1	34.0	35.7
Yes	107	62.6	72.0	46.9	54.9	66.0	64.3
Insurance Type							
Health insurance provided by employer	32	30.2	27.6	36.7	28.6	30.0	37.5
Health insurance purchased myself	5	4.7	5.3	3.3	3.6	5.7	0.0
Medicaid, Medicare or other governmental agencies	62	58.5	60.5	53.3	67.9	57.1	37.5
Other	7	6.6	6.6	6.7	0.0	7.1	25.0
Health Status							
Excellent	25	14.6	16.8	10.9	7.8	17.9	14.3
Very Good	39	22.8	20.6	26.6	19.6	23.6	28.6
Good	44	25.7	23.4	29.7	29.4	24.5	21.4
Fair	49	28.7	30.8	25.0	31.4	26.4	35.7
Poor	14	8.2	8.4	7.8	11.8	7.6	0.0
Triage level (*p* < 0.05)							
3	105	61.76	65.1	56.3	**72.6**	**53.3**	**85.7**
4	60	35.29	32.1	40.6	**25.5**	**43.8**	**7.1**
5	5	2.94	2.8	3.1	**2.0**	**2.9**	**7.1**

Note: values in bold are statistically significant at 0.05 level.

### Respondents’ ED visit characteristics and health care utilization

Respondents reported the main reasons for going to the ED were an accident or injury (*n* = 32; 18.7%), a new health problem (*n* = 46; 26.9%), and an ongoing condition or concern (*n* = 71; 41.5%). The top two reasons respondents used the AUMC ED were better customer service than other local EDs (*n* = 52; 30.4%) and vicinity (*n* = 37; 21.6%). Slightly more than half of the respondents traveled <10 miles (*n* = 95; 55.6%), and only 22 (12.9%) traveled >31 miles. More than half of the respondents (*n* = 108; 63.5) waited <5 min to be processed into the ED queue. More than half of the respondents (*n* = 105; 61.4%) reported they currently have a doctor’s office, clinic, or other place they usually go when they need a checkup, want advice about a health problem, or get sick or hurt. However, less than half of the respondents (*n* = 73; 42.7%) reported visiting a healthcare provider, not an ED, in the last 30 days. Only 31 (42.5%) saw their healthcare provider for the same reason they decided to go to the ED. Over one-third of the respondents (*n* = 64; 37.4%) reported not visiting the ED in the last 6 months. Among the respondents who reported being uninsured, one-fourth (*n* = 16; 25.0%) said they would use cash or credit, and slightly less than half (*n* = 26; 40.6%) said they were unsure how to pay the ED visit. We observed statistically significant differences for race by reason for choosing the AUMC ED (*p* = 0.06) with white participants choosing the AUMC ED mostly for an ongoing condition, distance traveled (*p* < 0.5) with black participants traveling <10 miles and white participants traveling >1 miles, and the last visit to their provider (*p* < 0.05) with white participants reporting being at the ED for the same medical reason. We observed statistically significant differences for gender with female participants reporting currently having a doctor’s office, clinic, or other place to go if needed (*p* < 0.05) and by visiting a healthcare provider, not an ED, in the past 30 days (*p* < 0.05) more often than male participants. Table 2 provides detailed information on the characteristics of the visit to the ED and the respondent’s health care utilization.

**Table 2.** Respondents’ Emergency Department Visit Characteristics and Health Care Utilization

Variable	*N*	%	Female %	Male %	White %	Black %	Other %
Thinking about this visit, what was the main reason why you came to the emergency room (ER)? (*p* = 0.06)							
A new health problem	46	26.9	24.3	31.3	25.5	29.3	14.3
An accident or injury	32	18.7	18.7	18.8	**9.8**	**23.6**	**14.3**
An ongoing condition	71	41.5	40.2	43.8	**51.0**	**37.7**	**35.7**
Other	22	12.9	16.8	6.3	**13.7**	**9.4**	**35.7**
Why did you select to come to this ER? Please select top two reasons							
Better customer service	52	30.4	26.2	37.5	39.2	27.4	21.4
Insurance coverage	11	6.4	8.4	3.1	3.9	5.7	21.4
Have a doctor who works at this hospital	35	20.5	21.5	18.8	21.6	19.8	21.4
Quicker service	11	6.4	7.5	4.7	5.9	7.6	0.0
Nearest ER	37	21.6	20.6	23.4	13.7	25.5	21.4
Other	25	14.6	15.9	12.5	15.7	14.2	14.3
Approximately, how many miles did you travel to arrive to this ER? (*p* < 0.05)							
less than 10	95	55.6	62.1	37.9	**15.8**	**77.9**	**6.3**
11–20	37	21.6	64.9	35.1	**43.2**	**43.2**	**13.5**
21–30	17	9.9	58.8	41.2	**47.1**	**47.1**	**5.9**
Over 31	22	12.9	63.6	36.4	**54.6**	**36.4**	**9.1**
When you first arrived to the ER, how long was it before someone asked you about the reason you came?							
less than 5 min	108	63.5	60.8	68.3	60.8	63.8	71.4
5–15 min	43	25.3	28.0	20.6	35.3	21.0	21.4
more than 15 min	19	11.2	11.2	11.1	3.9	15.2	7.1
Not counting the ER, is there currently a doctor’s office, clinic, or other place that you usually go if you need a checkup, want advice about a health problem, or get sick or hurt? (*p* < 0.05)							
No	66.0	38.6	**31.8**	**50.0**	27.5	43.4	42.9
Yes	105.0	61.4	**68.2**	**50.0**	72.6	56.6	57.1
In the last 30 days, have you visited a healthcare practitioner or provider (not ER)? (*p* < 0.05)							
No	98	57.3	**48.6**	**71.9**	56.9	58.5	50.0
Yes	73	42.7	**51.4**	**28.1**	43.1	41.5	50.0
Were the visits in the last 30 days to a healthcare practitioner or provider related to the reason you are here today? (*p* < 0.05)							
No	35	48.0	49.1	44.4	**31.8**	**61.4**	**14.3**
Not sure	7	9.6	7.3	16.7	**0.0**	**11.4**	**28.6**
Yes	31	42.5	43.6	38.9	**68.2**	**27.3**	**57.1**
In the last 6 months, how many times have you visited any ER to get care for yourself?							
none	64	37.4	33.6	43.8	43.1	34.9	35.7
1 time	32	18.7	19.6	17.2	15.7	20.8	14.3
2 times	34	19.9	20.6	18.8	13.7	20.8	35.7
3 times	17	9.9	8.4	12.5	11.8	9.4	7.1
over 4 times	24	14.0	17.8	7.8	15.7	14.2	7.1
If uninsured, how will you be paying for today’s ER visit?							
Not going to pay	3	4.7	6.7	2.9	4.4	5.6	0.0
Not sure	26	40.6	43.3	38.2	39.1	41.7	40.0
Will pay myself	16	25.0	16.7	32.4	17.4	30.6	20.0
Other	19	29.7	33.3	26.5	39.1	22.2	40.0

Note: values in bold are statistically significant at 0.05 level except for the first measure in the table, which has a *p* value of 0.06.

### Smartphone/tablet ownership and technical literacy

Respondents reported currently using a smartphone like an iPhone or Samsung (*n* = 139; 81.3%), a tablet like iPad, Kindle, or Samsung (*n* = 33; 19.3%), or a home computer with internet access (*n* = 33; 19.3%). Only a few of the respondents (*n* = 21; 12.3%) reported not having any internet-capable device. Respondents used video conferencing technologies such as FaceTime (*n* = 96; 56.1%), Skype (*n* = 42; 24.6%), Google Hangouts (*n* = 29; 17.0%), WhatsApp (*n* = 14; 8.2%), and Viber (*n* = 6; 3.5%). More than half of the respondents (*n* = 115; 67.3%) said they would have a caregiver or family member living with them who could help using the smartphone or tablet if needed. Respondents identified themselves as being very proficient with using a smartphone or tablet (*n* = 116; 67.8%), needing assistance with some applications (*n* = 29; 17.0%), needing assistance with most applications (*n* = 11; 6.4%), and never having used a smartphone or internet accessible device before (*n* = 15; 8.8%).

### Knowledge about telehealth

Most respondents (*n* = 148; 86.5%) had never heard about telehealth. Of those who had heard of telehealth (*n* = 23; 13.5%), only seven (30.4%) respondents used a telehealth consultation before, with six of them (85.7%) reporting using it to seek advice for mental health concerns and one (14.3%) to seek advice from a primary care doctor. Among the seven respondents who had used telehealth before, five (71.4%) were satisfied with the experience, and two (28.6%) were dissatisfied.

### Willingness-to-use and willingness-to-pay for telehealth

**FIG. 2.**
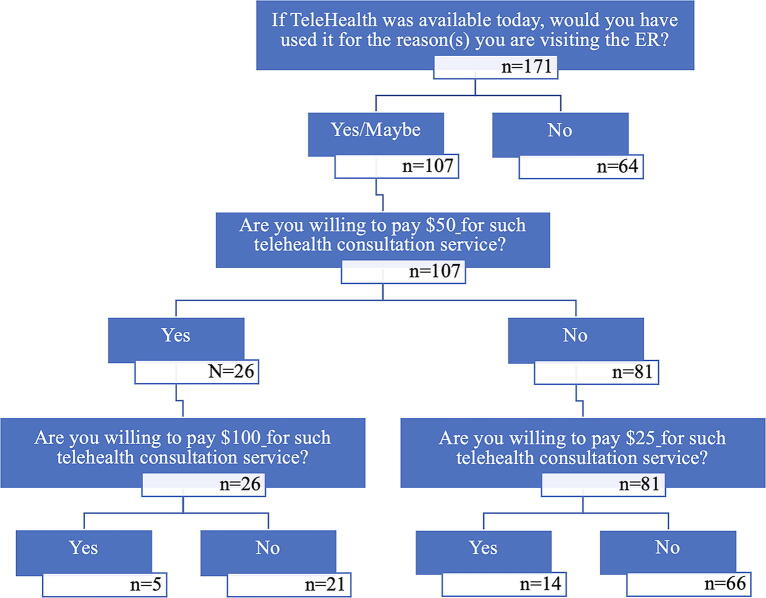
Willingness-to-use and willingness-to-pay study diagram and respondent responses.

After introducing the respondents to a hypothetical scenario where they could visit a board-certified emergency doctor by connecting to a telehealth consultation using their smartphone, tablet, or personal computer instead of coming to the ED, 54 (31.6%) of the respondents reported they would use such telehealth consultation, 64 (37.4%) would not, and 53 (31.0%) were unsure about using it. Only five (2.9%) of the respondents were willing to pay $100 for the telehealth consultation, 26 (15.2%) were willing to pay $50, and 14 (8.2%) were willing to pay $25. Figure 1 details the willingness-to-use and willingness-to-pay for telehealth questions, while Figure 2 shows the diagram of the question and the respondents’ responses.

We did not observe any statistically significant differences in a Chi-square test for the willingness-to-use by gender, race, insurance status, triage level, distance traveled, education level, employment status, the number of times the subjects visited the ED in the last 6 months, and by having a regular doctor/clinic. We also run a multivariable logistic regression model to estimate the associations between various sociodemographic factors (race, gender, triage level, age, education, marital status, health insurance, miles driven to the ED, and employment) and willingness-to-use telehealth. Our results indicate no statistically significant differences in respondents’ willingness-to-use based on their sociodemographic characteristics (Table 3).

**Table 3.** Multivariable Logistic Regression Model to Estimate the Associations Between Various Sociodemographic Factors and Willingness-to-Use and Willingness-to-Pay $50 for Telehealth

Variable	Willingness-to-use	Willingness-to-pay $50
Gender (ref. Female)		
Male	0.902	3.048^**^
Race (ref. White)		
Black	1.566	0.463
Other	2.577	0.471
Age (ref. 18–25)		
26–35	0.71	0.762
36–45	1.017	1.606
over 46	0.955	2.233
Education (ref. Less than High School)		
HS or GED	0.878	4.439
More than HS	0.645	2.734
Marital Status (ref. Single)		
Married/Partnered	0.945	1.89
Employment (ref. Employed)		
Unemployed	0.821	0.096^**^
Other	1.652	1.173
Insured (ref. None)		
Yes	0.805	0.190^**^
Miles traveled (ref. <10 miles)		
Over 10 miles	1.001	0.434

^**^ *p* < 0.05.

We also did not observe any statistically significant differences in a Chi-square test in the willingness-to-pay by race, triage level, distance traveled, education level, employment status, or the number of times the subjects visited the ED in the last 6 months. However, we observed statistically significant differences in the willingness*-*to-pay $50 by gender (*p* < 0.01), with men (40%) being more willing to pay than women (15%), for the willingness*-*to-pay $50 by currently having a regular doctor/clinic (*p* < 0.05) with those having a regular doctor/clinic (18%) being less willing to pay than those who do not have a regular doctor/clinic (35%), and for the willingness-to-pay $50 by health insurance status (*p* < 0.05) with those who have health insurance (16.4%) being less willing to pay than those who did not (37.5%). We also run a multivariable logistic regression model to estimate the associations between various sociodemographic factors and willingness-to-pay $50 out of pocket for telehealth. Our results showed that men were three times more likely to pay $50 for telehealth service than women (OR = 3.05; *p* = 0.04), individuals with health insurance were less likely to pay (OR = 0.19; *p* = 0.02) than individuals without any insurance and that individuals who were unemployed were less likely to pay (OR = 0.09; *p* = 0.02) compared to individuals who were employed (Table 3).

The major implications of our study are 1) hospitals should investigate telehealth as an option for nonlife-threatening ED visits and 2) educational initiatives are needed to increase public awareness of telehealth, and its benefits as technology does not appear to be a barrier.

## Discussion

Patient evaluation by emergency physicians via a telehealth system (audio and video) has been found feasible, safe, readily accepted by patients and providers, and associated with reduced throughput time.^18^ As telehealth infrastructure becomes more accessible and efficient, many medical centers have included telehealth in their clinical practice. By implementing access to telehealth from rural communities, patients can be given specialist care and close monitoring of health issues without the need for hospitalization or in-patient services.^19^ Hospital-based telehealth applications present a potentially significant solution, particularly for small and rural hospitals where access to local specialists is rarely available.^20^ Patients are interested in using their mobile devices for various health care-related functions at the point of care and beyond.^21^ For nonrural, relatively mobile patients, videoconferencing appears suitable for short visits for relatively simple complaints.^22^

When introducing older patients to technology in healthcare, it is essential to emphasize the possible benefits and offer support targeting their knowledge, skills, and motivation.^23^ Our study highlighted the telehealth readiness among the population under investigation regarding technology quality, access, and literacy. Similarly, a previous study investigating patient experiences and willingness-to-pay for cardiac telerehabilitation reported that most respondents (91%) possessed a smartphone and used it regularly to send text messages.^24^ However, the lack of knowledge and previous use of telehealth emphasize the importance of educating the population on telehealth opportunities.^25^

A previous research study indicated that patients farther from the healthcare provider were willing to use video conferencing to manage chronic diseases.^26^ Another study reported that highly motivated patients were open to shifting certain parts of cardiac telerehabilitation from face-to-face interactions to digital interactions.^24^ An encouraging result of our research was the high willingness-to-use telehealth after a brief description of telehealth was offered. A similar study in 2019 reported that 92% of participants were willing to utilize telehealth and would not go to the ED if they were determined low risk for emergency.^27^ Hospital administrations can decide to invest and provide telehealth services in their ED. It is essential to highlight that we did not observe any patients’ characteristics that would predict their willingness-to-use telehealth, making telehealth a service that is usable to anybody.

Willingness-to-pay research offers insights into the potential benefits of programs that intend to promote the growth of online health services. A previous research study reported that patients are willing to pay the same amount for a cardiac telerehabilitation session as a center-based session.^24^ Another study based on a US survey estimated the representative household was willing to pay $US 4.39 per month to receive remote diagnosis, treatment, monitoring, and consultations online for telehealth. Households living more than 20 miles from their nearest medical facility reported being willing to pay $US 6.22 per month.^28^ Because there were no statistically significant differences in respondents’ willingness-to-use based on their sociodemographic characteristics in our study, a single approach to improve respondent willingness-to-pay may be practical for all sociodemographic groups. Patient education to raise awareness on the benefits of telehealth consultations could increase the willingness-to-pay.^25^

A systematic review of the literature on the use of telehealth in ED reported strong evidence that telehealth positively impacts patient care while offering cost reduction for both hospital and patient.^29^ In our study, 40 (23.4%) respondents were willing-to-pay for a telehealth service. This service can target insured and uninsured people, possibly charging a higher premium to those with insurance. Insurance companies would still be willing-to-pay for such telecare services because such services would be significantly cheaper than paying for ED visits. While hospitals would not be able to recover all the costs of the telecare service billed to those patients without any insurance, hospitals will still be able to save money by not offering the services at their ED. This approach would also help hospital administrations with the issue of overcrowded EDs.

This study has limitations. We were able to recruit 171 patients; therefore, we cannot generalize our results to the ED patient population at large. Selection bias was introduced because patients who, despite being in a nonurgent triage level, self-reported feeling too unwell to participate in this study. We were not allowed to have graduate students present in the ED waiting room to collect data during the late evenings and the nights. It is possible that the willingness-to-use and willingness-to-pay would be different for patients visiting the ED after hours. The engagement of the participants could have been negatively affected by the absence of a private area in the ED waiting room where the survey could be taken.

## Conclusion

Even if our study indicated that many respondents had not heard about telehealth, they reported a generally positive attitude after reading a brief description. Considering many patients have access to health insurance, are technology literate, and have access to broadband connection either at home or on their mobile phones, offering telehealth services for nonemergent medical needs should be investigated further. Future studies should increase the number of subjects by reaching out to patients after they have visited the ED. The willingness-to-pay question should be asked only to the subjects who report not having any insurance coverage to avoid confusion among insured subjects who may think their insurance would not pay for such telecare service. Legal implications related to the use of telecare for nonemergent medical needs should also be investigated. A large national sample would help identify the characteristics of potential users of telecare services for nonemergent medical needs. Once the characteristics are determined, targeted advertisements could help increase knowledge and awareness of using telehealth instead of visiting the ED.

## Abbreviations Used

AUMC: Augusta University Medical Center
CVM: Contingent Valuation Method
DBDC: Double Bounded Dichotomous
ED: Emergency Departments
ESI: Emergency Severity Index
GED: General Educational Diploma
HS: High School
